# Differences in bioactivity between human insulin and insulin analogues approved for therapeutic use- compilation of reports from the past 20 years

**DOI:** 10.1186/1758-5996-3-13

**Published:** 2011-06-29

**Authors:** Haim Werner, Ernst A Chantelau

**Affiliations:** 1 Department of Human Molecular Genetics and Biochemistry, Sackler School of Medicine, Tel Aviv University, Tel Aviv 69978, Israel; 2 Formerly Heinrich-Heine-University of Düsseldorf/Germany, Holthorster Weg 16, 28717 Bremen, Germany

## Abstract

In order to provide comprehensive information on the differences in bioactivity between human insulin and insulin analogues, published *in vitro *comparisons of human insulin and the rapid acting analogues insulin lispro (Humalog^®^), insulin aspart ( NovoRapid^®^), insulin glulisine (Apidra^®^), and the slow acting analogues insulin glargine (Lantus^®^), and insulin detemir (Levemir^®^) were gathered from the past 20 years (except for receptor binding studies). A total of 50 reports were retrieved, with great heterogeneity among study methodology. However, various differences in bioactivity compared to human insulin were obvious (e.g. differences in effects on metabolism, mitogenesis, apoptosis, intracellular signalling, thrombocyte function, protein degradation). Whether or not these differences have clinical bearings (and among which patient populations) remains to be determined.

## Introduction

Since the first insulin derivatives were synthetised in the 1970s for scientific purposes [1,2], the therapeutic potential of these compounds for diabetic patients was already under investigation. In those times, there was a quest for metabolically superactive insulins, i.e. "tailor-made" insulin derivatives with enhanced biopotency, like the insulin derivative B10Asp [3], to make the treatment of diabetes mellitus more efficacious [3]. And B10Asp was indeed prepared to be marketed. In 1992 (when the carcinogenicity of B10Asp [4] was disclosed by haphazard) it was realized that manipulations of the insulin molecule could introduce the risk of artificial bioactivities into the treatment of diabetic patients [4-6]. Probably because of such concerns, insulin manufacturers subsequently favoured the design of derivatives (euphemistically called insulin analogues) with particular absorption properties (i.e. faster or more prolonged absorption from the subcutaneous tissue after injection [7,8]). Since 1996, five of such insulin analogues have been approved for human use (the rapid acting analogues aspart, glulisine and lispro, and the slow-acting analogues glargine and detemir). Their biological potencies still remain to be fully elucidated [4]. In order to expose the known differences in all fields of bioactivity between these insulin analogues and human insulin, we registered all *in vitro *studies - except for receptor binding studies - published from 1990 to 2010 and displayed their findings schematically in the following report.

## Materials and methods

An electronic search was conducted in the PubMed database using the following key words: *in vitro*, proliferation, mitogenic, mitogenicity, metabolic, apoptosis, potency, glucose transport, lipogenesis, intracellular signalling, in conjunction with the currently marketed insulin analogues searched for by their international non-proprietary names: insulin aspart [B28Asp human insulin] (NovoNordisk, Bagsvaerd, Denmark), insulin detemir [B29Lys(epsilon-tetradecanoyl), desB30 human insulin] (NovoNordisk, Bagsvaerd, Denmark), insulin glargine [A21Gly,B31Arg,B32Arg human insulin] (Sanofi-Aventis, Paris, France), insulin glulisine [B3Lys,B29Glu human insulin] (Sanofi-Aventis, Paris, France) and insulin lispro [B28Lys,B29Pro human insulin] (Eli Lilly, Indianapolis, IN, USA). Their respective brand names Humalog ^®^, NovoRapid^®^, Lantus^®^, Apidra^®^, Levemir^® ^and their company abbreviations HOE 901(= Lantus^®^), NN 304(= Levemir^®^), HMR 1964 (= Apidra^®^), B28Asp (= NovoRapid^®^) and LY275585(= Humalog ^®^) were also searched for. A hand search was performed on reference lists, published symposium reports, and abstract volumes of scientific meetings. The time span covers the years 1990 to 2010. Receptor binding studies were not addressed in this search. The publications are reported in a schematic fashion, in alphabetical order of the analogues' international non-proprietary names.

## Results

Altogether, 50 publications were retrieved reporting *in vitro *data on comparisons between human (native) insulin and one of the insulin analogues indicated above [5,9-57]. There were 45 full papers [5,9,21,23-27,31-38,40-57] and 5 abstracts [22,28-30,39]. The publications were screened for differences in seven categories: metabolic activity, mitogenic activity, anti-apoptotic activity, intracellular signalling, effects on thrombocytes, effects on protein degradation, and intracellular internalization. The studies were performed or sponsored by pharmaceutical companies (n = 27) or independent institutions [10,12,16-18,27,31-34,37,39,41-43,55-57]; in 3 studies, possible sponsoring could not be identified.

### Differences in metabolic activity

There were 14 publications reporting *in vitro *studies on the metabolic activity of insulin analogues (assessed in primary mouse adipocytes, primary rat cardiomyocytes and adipocytes, human muscle cells, human dermal microvascular endothelial cells, 3T3-L1 adipocytes, and L6 myocytes) in comparison to synthetic human insulin.

#### Aspart

This issue was addressed in the Scientific Discussion of insulin aspart, published by the European Medicines Agency (EMEA) (page 4: "in mouse free fat cells, the stimulation of lipogenesis did not differ between insulin aspart and human insulin, lending further support to similar molar potency " [58]). In primary rat adipocytes, the effects on glucose transport and lipogenesis were similar to human insulin [21], and in primary mouse adipocytes the effect on lipogenesis was similar to human insulin [26,53].

#### Detemir

This issue was addressed in the Scientific Discussion of insulin detemir, published by the EMEA (pages 1-6: "insulin detemir was consistently less potent than human insulin in all *in vitro *assays. Depending on the assay, the potency was 2- to 10-fold lower" [59]). In primary mouse adipocytes [26] and primary rat adipocytes [48], in 3T3-L1 adipocytes [12,54] and in L6 myocytes [54] the metabolic potency was only 1-20% of that of human insulin by equimolar comparison. (Therefore, the manufacturer increased the concentration of the active substance in the vial four-fold compared to synthetic human (or porcine natural) insulin [48]: 1 unit of human insulin is 6 nmol insulin, while 1 unit of insulin detemir is 24 nmol [59]).

#### Glargine

This issue was addressed in the Scientific Discussion of insulin glargine, published by the EMEA (page 5: "nearly all *in vitro *metabolic studies have shown a relative *in vitro *potency for insulin glargine of about 50% and similar maximal responsiveness as compared to human insulin....there is evidence that higher plasma levels *in vivo *compensate for the 50% *in vitro *potency found for insulin glargine as compared to human insulin"[60]). In primary mouse adipocytes [26], primary rat cardiomyocytes [9,46] and in 3T3-L1 adipocytes [19,25] metabolic potency was only about 50% of that of human insulin by equimolar comparison. Another study in 3T3L1 adipocytes showed a similar metabolic potency to human insulin [55]. In non-starved muscle cells, the effect on glucose uptake was similar to human insulin [14]. In human dermal microvascular endothelial cells, there was no effect, similar to human insulin [13].

#### Glulisine

This issue was addressed in the Scientific Discussion of insulin glulisine, published by the EMEA (page 8: "Stimulation of glucose transport was equal for both insulin glulisine and human insulin...lipogenic activity and glucose transport in isolated rat adipocytes was slightly lower than human insulin, but reached the same maximum obtainable effects as human insulin at higher concentrations"[61]). In rat cardiomyocytes, the effect on glucose transport [35], and in non-starved muscle cells, the effect on glucose uptake [15] was similar to human insulin.

#### Lispro

This issue was addressed in the Scientific Discussion of insulin lispro, published by the EMEA (page 2: "Insulin lispro is biologically equivalent to insulin in several *in vitro *tests, including ...glucose transport in adipocytes" [62]). In primary mouse adipocytes [26] the effect on lipogenesis was comparable to that of human insulin. A study in L6 myotubes showed a similar metabolic potency compared to human insulin [47].

### Differences in mitogenic activity

As standard 2-year carcinogenicity *in vivo *studies in animals (see EMEA: Note for Guidance on Carcinogenic Potential; CPMP/SWP/2877/00) have not been performed with any of the commercially available insulin analogues (except for insulin glargine [58-62]), the issue of mitogenicity has been addressed by quite a few *in vitro *investigations. There were 34 publications reporting *in vitro *data on the mitogenic activity of insulin analogues with various non-malignant cell types: primary muscle cells, human smooth muscle cells, Chinese hamster ovary cells (CHO-K1), human dermal microvascular endothelial cells, human mammary epithelial cells, rat-H9c2 myoblasts, H9 cardiac myoblasts, rat-1 fibroblasts, rat H4-II-E hepatoma cells, K6 myoblasts, L6 myoblasts, HepG2 cells, C2C12 myoblasts, mammary gland-derived MCF-10 cells, human coronary artery endothelial cells, human coronary artery smooth muscle cells, primary human smooth muscle cells, vascular smooth muscle cells, primary mouse mammary gland epithelial cells, human lung fibroblast HEL-299 cells, and rat thyrocytes FRTL-6. Various malignant cell types were also studied: human osteosarcoma cells (Saos/B10 cell, Saos-2 cells), human mammary carcinoma cells (MCF-7 cells, T47 D cells, MDA-MB 231 cells, SKBR-3 cells), human colonic carcinoma cells HCT-116, pancreatic carcinoma cells Colo-357, prostate carcinoma cells PC-3, thyroid cancer cells FTC-133, and human bladder cancer cells T 24. The experiments were performed in comparison with synthetic human insulin.

#### Aspart

This issue was addressed in the Scientific Discussion of insulin aspart, published by the EMEA (page 4: "The results in CHO K1 cells were essentially similar to those of human insulin whereas the mitogenic activity of insulin aspart in MCF-7 cells indicated differences to human insulin. Subsequent analysis indicated lower activity than initially calculated but the analysis also showed that the results in MCF-7 cells were not sufficiently robust for proper assessment. Newly performed studies using human osteosarcoma B10 cells revealed essentially similar response of insulin aspart and human insulin" [58]).

In four studies using non-malignant cells (muscle cells, CHO-K1 cells, MCF-10 A cells) insulin aspart showed a similar mitogenic potency, compared to human insulin [5,21,32,41,42]. In malignant MCF-7 cells and T 47 D cells, insulin aspart showed similar mitogenic potency as human insulin [32]. In malignant Saos/B10 cells, insulin aspart showed lower mitogenic potency [26]. In one study using the malignant MCF-7 cells, insulin aspart showed a non-significantly higher mitogenic potency compared to human insulin [41,42].

#### Detemir

This issue was addressed in the Scientific Discussion of insulin detemir, published by the EMEA (page 8: "Relative to human insulin, the albumin-corrected mitogenic potency was 9% in CHO-K1 cells, 15% in a human mammary cancer cell line (MCF-7 cells), 11% in a human osteosarcoma cell line, and 25% in L6-IhR cells. Based on these data, the mitogenic potency of insulin detemir *in vitro *seems to be reduced relative to human insulin to approximately the same extent as its binding affinity for the insulin and IGF-1 receptors" [59]). Ten years later, however, the company emphasized that "the potency estimates are different between the *in vivo *and *in vitro *conditions" [48]. There were nine studies which had equimolar comparisons to human insulin, and one that was based on equipotent comparison [33].

In five studies using non-malignant cells (vascular smooth muscle cells [54], MCF-10A cells [32,41,42], HEL-299 cells [55]), insulin detemir showed similar mitogenic potency compared to human insulin. In one study using non-malignant human muscular endothelial cells [22] and L6-hlR cells [22], the mitogenic potency of insulin detemir was non-significantly lower compared to human insulin. Using the malignant MCF-7 cells, insulin detemir showed similar mitogenic potency in two studies [32,41,42], and higher mitogenic potency in one study [56]. Studies using the malignant Saos/B10 cells [26], and T24 cells [30] showed less mitogenic potency of insulin detemir, and a study using HCT-116 cells [56] and PC-3 cells [56] showed higher mitogenic potency of insulin detemir compared to human insulin. In the one study using MCF-7 cells with equipotent comparison, insulin detemir showed similar mitogenic potency compared to human insulin [33].

#### Glargine

This issue was addressed in the Scientific Discussion of insulin glargine, published by the EMEA (page 7: "...insulin glargine might have a mitogenic potential through binding to the IGF-1 receptor. This point of concern was addressed in an oral explanation at the Committee for Proprietary Medicinal Products (CPMP) where the company highlighted that insulin glargine had a lower mitogenic activity than the comparator B10-Asp insulin or IGF-1 in 3 out of 4 cell assays"[60]). There were 26 studies on this topic, all with equimolar comparisons to human insulin.

In 12 studies using non-malignant cells (H9 rat cardiomyoblasts [9,28], rat-1 fibroblasts [11], primary human muscle cells [14], primary human smooth muscle cells [16], human coronary artery smooth muscle cells [49], human coronary artery endothelial cells [49], vascular smooth muscle cells [54], MCF-10 A cells [32,41,42], rat thyrocytes FRTL-5 [34] and HepG2 cells [19]), insulin glargine showed similar mitogenic potency compared to human insulin. In 5 studies using non-malignant cells (human dermal microvascular endothelial cells [13], human mammary epithelial cells [22,25], individual primary human smooth muscle cells [16], HEL-299 cells [55]), insulin glargine showed higher mitogenic potency compared to human insulin. In one study using the non-malignant L6-hlR cells [22], insulin glargine showed lower mitogenic potency compared to human insulin.

In 6 studies using malignant cells (MCF-7 cells [50], Colo-357 cells [17], T47 D cells [32], MDA-MB-231 cells [29], T24 cells [31], thyroid cancer FTC-133 cells [34], insulin glargine displayed similar mitogenic potency as compared to human insulin. In 12 studies using malignant cells (Saos/B10 cells [21,26,39,46], MCF-7 cells [27,32,33,41,42,56], SKBR-3 cells [27], HCT-11 cells [56] and PC-3 cells [56], insulin glargine showed increased mitogenic potency compared to human insulin. In one study using Saos/B10 cells, the insulin glargine metabolite IM showed increased mitogenic potency [46], while the glargine metabolites M1 and M2 showed similar mitogenic potency compared to human insulin [46].

#### Glulisine

This issue was addressed in the Scientific Discussion of insulin glulisine, published by the EMEA (page 9: "(thymidine incorporation) was equal for insulin glulisine and human insulin.." [61]).

Six studies using non-malignant cells (K6 myoblasts [35], C2C12 myoblasts [23], primary human muscle cells [15], rat-1 fibroblasts [51], MCF-10 A cells [32,43] and mammary gland cells [43]) showed equal mitogenic potency of insulin glulisine and human insulin, whereas one study using MCF-10 cells showed less mitogenic potency of insulin glulisine [24].

Two studies in malignant MCF-7 cells [32,43] showed equal mitogenic potency of insulin glulisine and human insulin.

#### Lispro

This issue was addressed in the Scientific Discussion of insulin lispro, published by the EMEA (page 2: "In cell growth assays using human smooth muscle cells and human mammary epithelial cells and using... thymidine incorporation or increases in cell number as index of cell growth, insulin lispro was shown to be equipotent to human insulin"[62]).

In five studies using non-malignant cells (human mammary epithelial cells [45], rat H4-II-E hepatoma cells [20], and MCF-10A cells [32,41,42], insulin lispro displayed similar mitogenic potency compared to human insulin. In one study using human smooth muscle cells [44], insulin lispro displayed higher mitogenic potency, and in another study using human smooth muscle cells, insulin lispro displayed lower mitogenic potency compared to human insulin [44].

In three of the studies using malignant cells (MCF-7 [32,41,42] and T 24 D [32]), insulin lispro displayed equal mitogenic potency compared to human insulin; in one study using Saos/B10 cells [26], insulin lispro showed lower, and in one study using HCT-116 cells [56], insulin lispro showed higher mitogenic potency compared to human insulin.

### Differences in anti-apoptotic activity

This issue was not specifically addressed in the Scientific Discussions of any of the five analogues published by the EMEA [58-62]). However, there were six publications [17,36,49,52,56,57] reporting *in vitro *data on the inhibition of apoptosis by insulin analogues versus human insulin.

#### Aspart

There was only one study of insulin aspart using malignant INS-1 cells [36]; the inhibition of apoptosis was similar compared to human insulin.

#### Detemir

There were two studies of insulin detemir, conducted with equimolar comparisons to human insulin in malignant HCT-116 cells [56,57]; in both studies, insulin detemir inhibited apoptosis more than did human insulin.

#### Glargine

There was one study using non-malignant cells (human coronary endothelial cells [49] and human coronary artery smooth muscle cells [49]) showing no effect on apoptosis by either insulin glargine or human insulin. In one study conducted with equimolar concentrations of insulin glargine and human insulin in malignant Colo-357 cells [17], inhibition of apoptosis was similar between both compounds. In malignant HCT-116 cells [56,57], and malignant INS-1 cells [52], insulin glargine inhibited apoptosis stronger compared to human insulin.

#### Glulisine

There were two studies of insulin glulisine, conducted in malignant INS-1 cells [36,52], showing stronger inhibition of apoptosis by insulin glulisine compared to human insulin.

#### Lispro

There were two studies of insulin lispro, conducted in malignant INS-1 cells [36,52], one showing similar inhibition [52], and one showing stronger [36] inhibition of apoptosis by insulin lispro compared to human insulin.

### Differences in intracellular signaling

The issue was not specifically addressed in the Scientific Discussions of any of the five analogues published by the EMEA [58-62]. However there were 12 publications [14,23,31,35,40-43,47,54,56,57] reporting *in vitro *data on intracellular signalling by insulin analogues versus human insulin concerning the Akt-GSK-3 pathway (involved in metabolic activity), and the Erk-MAPK pathway (involved in proliferative activity).

#### Aspart

There were two studies of insulin aspart, conducted in non-malignant MCF-10A cells and malignant MCF-7 cells [41,42], and in IGF-1 receptor-deprived mouse fibroblasts transfected with either the human insulin receptor A or B isoforms [40], all of which showed equal Akt-activation and GSK-3 inactivation of insulin aspart compared to human insulin. Erk 1/Erk 2 phosphorylation by insulin aspart was similar to human insulin in MCF-10A cells [41,42], but was increased in fibroblasts expressing insulin receptor A isoform [40], and decreased in fibroblasts expressing insulin receptor B isoform [40].

#### Detemir

There were five studies of insulin detemir conducted with equimolar comparisons to human insulin [41,42,54,56,57], and one with equipotent comparison [40].

A similar Akt-activation by insulin detemir and human insulin was found in non-malignant MCF-10A cells [41,42] and in non-malignant fibroblasts expressing insulin receptor A isoform [40]. A weaker Akt-activation by insulin detemir compared to human insulin was found in non-malignant cells (3T3-L1 adipocytes [54], L6 myocytes [54], primary hepatocytes [54], primary vascular smooth muscle cells [54]), and in non-malignant fibroblasts expressing insulin receptor B isoform [40].

In non-malignant cells (3T3-L1 adipocytes [54], L6 myocytes [54], primary hepatocytes [54], primary vascular smooth muscle cells [54]) GSK-3 inactivation by insulin detemir was weaker than that by human insulin. In non-malignant MCF-10A cells, GSK-3 inactivation was similar by insulin detemir and human insulin [41,42]. Erk 1/Erk 2 phosphorylation by insulin detemir was equal to that by human insulin in two studies using non-malignant cells (MCF-10A cells [41,42] and fibroblasts expressing insulin receptor B isoform [40]. Erk 1/Erk 2 phosphorylation was increased by insulin detemir in fibroblasts expressing insulin receptor A isoform [40].

In non-malignant cells (3T3-L1 adipocytes [54], L6 myocytes [54], primary hepatocytes [54], primary vascular smooth muscle cells [54]), MAP-kinase activation by insulin detemir was weaker than that by human insulin. In malignant cells (MCF-7 [41,42], HCT-116 [56,57]), Akt-activation by insulin detemir was weaker than that by human insulin. In MCF-7 cells, GSK-3 inactivation was weaker by insulin detemir as compared to human insulin [41,42]. Erk 1/Erk 2 phosphorylation was decreased by detemir in MCF-7 cells [41,42] and HCT-116 cells [56,57]. In MCF-7 cells, MAP-kinase activation by detemir was similar to that by human insulin [41,42].

#### Glargine

There were eight studies of insulin glargine, all of which with equimolar comparisons to human insulin [14,31,40-42,54,56,57]. Akt-activation by insulin glargine was similar to that by human insulin in non-malignant primary human muscle cells [14], 3T3-L1 adipocytes [54], L6 myocytes [54], primary hepatocytes [54], primary vascular smooth muscle cells [54], and in fibroblasts expressing insulin receptor A isoform [40]. In non-malignant MCF-10A cells, Akt-activation by insulin glargine was stronger [41,42], and in fibroblasts expressing insulin receptor B isoform it was weaker than that by human insulin [40]. GSK-3 inactivation by insulin glargine was similar to that by human insulin in non-malignant 3T3-L1 adipocytes [54], L6 myocytes [54], primary hepatocytes [54], primary vascular smooth muscle cells [54], and MCF-10A cells [41,42]. Erk 1/Erk 2 phosphorylation by insulin glargine was equal to that by human insulin in fibroblasts expressing insulin receptor B isoform [40], it was stronger in fibroblasts expressing insulin receptor A isoform [40], and weaker in MCF-10A cells [41,42]. MAP-kinase activation by glargine was equal to that by human insulin in primary human muscle cells [14] and in primary rat vascular smooth muscle cells [54].

#### Glulisine

Akt-activation by insulin glulisine was similar to that by human insulin in non-malignant rat cardiomyocytes [35], and in fibroblasts expressing insulin receptor A isoform [40]. Akt-activation by insulin glulisine was weaker than that by human insulin in MCF-10A cells [43], in mouse muscle tissue [23], in mouse liver tissue [23], and in fibroblasts expressing insulin receptor B isoform [40]. GSK-3 inactivation by insulin glulisine was similar to that by human insulin in rat cardiomyocytes [35], and weaker in MCF-10A cells [43]. Erk 1/Erk 2 phosphorylation by insulin glulisine was similar to that by human insulin in MCF-10 cells [43], and weaker in K6 myoblasts [35], in fibroblasts expressing insulin receptor A isoform [40], and in fibroblasts expressing insulin receptor B isoform [40]. MAP-kinase activation by glulisine was similar to that by human insulin in mouse muscle tissue [23], and mouse liver tissue [23].

#### Lispro

Akt-activation by insulin lispro was similar to that by human insulin in non-malignant MCF-10 A cells [41,42], and in fibroblasts expressing insulin receptor A isoform [40]. Akt-activation by insulin lispro was weaker than that by human insulin in fibroblasts expressing insulin receptor B isoform [40]. GSK-3 inactivation by lispro was similar to that by human insulin in MCF-10A cells [41,42]. Erk 1/Erk 2 phosphorylation by insulin lispro was similar to that by human insulin in L6 myocytes [47], and in MCF-10A cells [41,42]; it was weaker in fibroblasts expressing insulin receptor B isoform [40], and stronger in fibroblasts expressing insulin receptor A isoform [40].

The studies mentioned above are listed in Table 1.

**Table 1:** Bioactivities of insulin analogues versus human insulin

		Aspart	Detemir	Glargine	Glulisine	Lispro
	lower		12,26,48,54	9,19,25,26		
	
**metabolic activity**	equal	21,26,53		13,14,55	15,35	26,5
	
	higher					

	lower	26	22,26,30	22		26,44
	
**mitogenic activity**	equal	5,21,32,41,42	32,33,41,42,54,55	9,11,14,16,17,19,28,29,31, 32,34,41,42,49,50,54	15,23,32,35,43,51	20,32,41,42,45
	
	higher	41,42	56	13,16,21,22,25-27,32,33,39, 41,42,46,55,56		44,56

	lower					
	
**apoptosis inhibition**	equal	36		17,49		52
	
	higher		56,57	52,56,57	36,52	36

	lower		40-42,54,56,57	40	23,40,43	40
	
**Akt-activation**	equal	40-42	40-42	14,40,54	35,40	40-42
	
	higher			41,42		

	lower		41,42,54	41,42,54	43	41,42
	
**GSK-3 inactivation**	equal	40-42	41,42		35	
	
	higher					

	lower	40	41,42,56,53	41,42	23,40,43	40
	
**Erk-phosphorylation**	equal	41,42	40-42	40	35,40	41,42,47
	
	higher	40	40	40		40

	lower		54			
	
**MAP-kinase activation**	equal		41,42	14,54	23	
	
	higher					

Compliation of reports (numbers refer to the publications cited in the reference list) on bioactivities of the insulin analogues aspart, detemir, glargine, glulisine, and lispro versus human insulin.

### Differences in effects on thrombocytes

The issue was not specifically addressed in the Scientific Discussions of any of the five analogues published by the EMEA [58-62]. However, there were two publications [37,38] that compared the effects of insulin analogues and human insulin on thrombocyte function.

#### Aspart

Inhibition of platelet aggregation was stronger than that by human insulin; both compounds affected platelet aggregation via PI-3 K activation [37].

#### Lispro

Inhibition of platelet aggregation was stronger than that by human insulin; both compounds affect platelet aggregation via PI-3 K activation [38].

### Differences in effects on protein degradation

This issue was not specifically addressed in the Scientific Discussion of any of the five analogues published by the EMEA [58-62]. However, there were two publications [18,19] on this topic.

#### Glargine

Inhibition of protein degradation in HepG2 cells was less than that by human insulin [19].

#### Lispro

Inhibition of protein degradation in H4-II-E hepatoma cells was much stronger than that by human insulin, and inhibition of protein degradation in Hep2G cells and in L6 cells was similar to that by human insulin [18].

### Differences in intracellular internalization and degradation

The issue was not specifically addressed in the Scientific Discussion of any of the five analogues published by the EMEA [58-62]. However, there were four publications [10,19,20,35] on this topic.

#### Glargine

Internalization into HepG2 cells was similar to that of human insulin, whereas degradation was much less compared to human insulin [19].

#### Glulisine

Internalization into and degradation by K6 rat myoblasts was less compared to human insulin [35].

#### Lispro

Internalization into rat hepatocytes was similar to that of human insulin [20]; degradation by insulin degrading enzyme (IDE) was similar to that of human insulin [10,20].

### Miscellaneous

#### Effect on gene expression

This issue was not specifically addressed in the Scientific Discussion of any of the five analogues published by the EMEA [58-62]. However, there were three publications [12,41,43] on this topic.

#### Detemir

In 3T3-L1 preadipocytes, the effect of insulin detemir on the gene expression of leptin and PPAR-Gamma-2 was considerably reduced compared to human insulin [12].

#### Glargine

In MCF-7 cells, insulin glargine induced a higher expression of the Cyclin D1 gene, compared to human insulin [41].

#### Glulisine

In HepG2 cells, neither insulin glulisine nor human insulin induced hexokinase-4 expression [43]. In HepG2 cells, hexokinase-2 expression was induced to the same extent by both 150 nmol human insulin and insulin glulisine; no effect of either compound at lower concentrations was observed in HepG2 cells or MCF-7 cells [43].

### Addendum: differences in bioactivity between native porcine or bovine insulin and synthetic human insulin

For comparison, the bioactivities of synthetic human insulin versus native animal insulin (bovine and porcine) are reported, as retrieved from six publications [32,41-43,63,64].

#### Metabolic potency

In human and rat adipocytes, porcine insulin showed slightly higher metabolic potency (in terms of lipogenesis in rat epididymal and human subcutaneous fat cells), as compared to synthetic (recombinant DNA) human insulin (Humulin^®^, Eli Lilly, Indianapolis, IN, USA) [64].

#### Mitogenicity

In MCF-7 cells, bovine insulin showed slightly less mitogenic potency compared to synthetic human insulin (Actrapid^®^, NovoNordisk, Bagsvaerd, Denmark), whereas in MCF-10A and T47 D cells, bovine insulin showed a similar mitogenic potency to human insulin [32,41-43]. In bovine dermal fibroblasts GM06034 and GM06035, bovine insulin and synthetic human insulin (Humulin^®^) displayed a similar mitogenic potency [63].

#### Intracellular signalling

In MCF-7 and MCF-10A cells, bovine insulin showed a slightly weaker Erk 1/2 phosphorylation compared to synthetic human insulin (Actrapid^®^), whereas the effect on Akt-phosphorylation and GSK 3 alpha/beta-phosphorylation was similar to that of synthetic human insulin [41-43].

#### Gene expression

In HepG2 cells, neither bovine nor human insulin induced hexokinase 4 expression [43].

## Discussion

Our schematic presentation shows that in the 1990s most *in vitro *studies were industry-sponsored ones (except for study [5]), and were performed using non-malignant cells, whereas in later years, the majority of studies were performed using malignant cells, and many of them were carried out by industry-independent investigators. This change in direction may have been brought about by the *Points to Consider *Document of the EMEA [65] of 2001, which particularly requested studies using malignant cell lines, or malignant tissues, or animal models of malignancies to prove the safety of insulin analogues in terms of tumour growth promotion. The rationale for this request was that non-malignant and malignant tissues may respond differently to stimulation by insulin or insulin analogues, due to differences in the expression (or the function) of insulin receptors and IGF-1 receptors between non-malignant and malignant tissues.

Moreover, we found that the metabolic bioactivity of insulin analogues was studied exclusively on benign primary fat cells, or fat cell lines. According to these investigations, the analogue insulins aspart, glulisine and lispro were shown to have equal metabolic potencies to human insulin, whereas the analogues glargine and detemir considerably reduced metabolic activity. The growth-promoting bioactivity, e.g. effects on mitogenicity and apoptosis, showed equal potencies of the insulin analogues and human insulin in most circumstances- when studied in benign cells. Different effects, particularly on mitogenicity and apoptosis, only became apparent when the insulin analogues and human insulin were studied in malignant cells. Intracellular signalling was examined for all of the insulin analogues, and considerable differences to human insulin were found in both non-malignant and malignant cells.

Of note are the considerable inconsistencies between the findings of various studies that sometimes seemed contradictory. These discrepancies were most likely due to methodological differences between the various study settings and designs, such as the following:

### The type of cells studied

It was shown that a high ratio of IGF-1 receptors to insulin receptors is required for a strong mitogenic effect of glargine [66]. It is likely that this applies not only to cell-lines, but also to primary cells, and to primary tumour cells and tissues. Considerable differences in receptor expression may exist between health and disease [67,68], and between individuals [16]. Many human cancers overexpress IGF-1 receptors, particular insulin receptors, and hybrid receptors 67, 69], in contrast to healthy tissues. Thus, cancer cell-lines or cancer tissue may be more susceptible to growth stimulation by insulin or insulin analogues than healthy tissue, and some cancer cell-lines may be more susceptible than others. Furthermore, metabolic potency studies may yield different results when performed in rat epididymal fat cells or in human subcutaneous fat cells [64].

### The type of culture medium used

Some authors used high glucose concentrations, and some used low glucose concentrations in the cell cultures. The concentration of glucose may have a significant effect on the growth of cells in culture [70]. Moreover, the presence or absence of albumin, or of fetal calf serum (FCS) affects cell growth and the susceptibility of cells to growth factors or insulin: 10% FCS stimulates cell growth as much as does 10 nmol/l IGF-1 [5,63]. The content of insulin, oestrogen and IGF-1, for example, in FCS is rarely indicated by the manufacturer, and may vary between batches. Thus, starved cell cultures may yield different results compared to FCS-fed (i.e. maximally growing) cells in terms of stimulation by insulin or IGF-1.

### Presence of albumin in the assay

The study data with detemir were particularly confusing because this analogue was sometimes compared to human insulin on an equimolar basis (which is inappropriate, because detemir in equipotent doses requires 4 times the molar concentration compared to human insulin). Moreover, in the presence of albumin the activity of detemir is dramatically reduced [54] and not all studies on detemir appropriately accounted for this phenomenon. Furthermore, the albumin species plays a role, for detemir binding to albumin differs according to the albumin species (i.e. it differs in dogs versus rabbits or pigs etc.). Hence, it could be argued that most of the previous *in vitro *studies comparing insulin detemir with other insulins are basically uninterpretable.

### The concentration and the EC50 value of the ligand under study

The concentrations of insulin and the insulin analogues played a major role in the outcome of the experiments - low concentrations and high concentrations may yield qualitatively different cell responses. A comparison of bioactivities based on EC50 values rather than on equimolar concentrations might be preferable.

### The duration of cell culture studies

The studies were carried out over varying time intervals, ranging from less than 1 hour to several days. In some studies, insulin or the insulin analogues were supplemented only once, whereas in other studies they were supplemented repeatedly (taking into account the fact that insulin degradation may occur in the culture). These aspects may have affected the performance of the assays.

The heterogeneity in methods and outcomes suggests that a standardization of the procedures for *in vitro *examinations of insulin analogues would be required in order to make the data more comparable. Whether these (and possible additional) *in vitro *differences in bioactivity between human insulin and the insulin analogues aspart, detemir, glargine, glulisine, and lispro translate into clinical outcomes remains to be elucidated. Patient populations with particular genetic make-up may be particularly susceptible [71]. There are already some indications that the *in vitro *differences might matter in clinical practice [33], and that other differences as disclosed by *in vivo *studies (e,g, glargine may increase serum IGF-1 concentrations in diabetic patients [72], detemir largely increases serum insulin levels [48,73] and may accumulate in the liver and the brain [74]) might matter, too.

In conclusion, the schematic presentation of the currently available *in vitro *data (except for receptor studies) on the differences in bioactivity between human insulin and the insulin analogues aspart, detemir, glargine, glulisine, and lispro displays a variety of abnormal activities of the analogues. The data- albeit suffering from heterogeneity between the assays-suggests that manipulation of the insulin molecule in order to improve its absorption from the subcutaneous tissue may cause unphysiological bioactivities with hitherto unknown consequences for the diabetic user as well as for the laboratory researcher.

## Competing interests

The authors declare that they have no competing interests.

## Authors' contributions

EAC did the search, EAC and HW participated in the screening and analysis of the data and in the writing of the final manuscript. All authors have read and approved the final version of the article.

## References

[B1] Brandenburg D Insulin-structure, function, design Exp Clin Endocrinol Diabetes 1999 107 Suppl 2 S6 S12

[B2] Ellis MJ Darby SC Jones RH Sönksen PH In vitro bioactivity of insulin analogues: lipogenic and anti-lipolytic potency and their interaction with the effect of native insulin Diabetologia 1978 15 403 410 10.1007/BF01219650 738550

[B3] Burke GT Hu SQ Ohta N Schwartz GP Zong L Katsoyannis PG Superactive insulin Biochem Biophys Res Commun 1990 173 982 987 10.1016/S0006-291X(05)80882-4 2268358

[B4] Eckardt K Eckel J Insulin analogues: action profiles beyond glycaemic control Arch Physiol Biochem 2008 114 45 53 10.1080/13813450801983369 18465358

[B5] Bornfeldt KE Gidlöf RA Wasteson A Lake M Skottner A Arnqvist HJ Binding and biological effects of insulin, insulin analogues and insulin-like growth factors in rat aortic smooth muscle cells. Comparison of maximal growth promoting activities Diabetologia 1991 34 307 313 10.1007/BF00405001 1713869

[B6] Tseng LY Schwartz GP Sheikh M Chen ZZ Joshi S Wang JF Nissley SP Burke GT Katsoyannis PG Rechler MM Hybrid molecules containing the A-domain of insulin-like growth factor-1 and the B-chain of insulin have increased mitogenic activity relative to insulin Biochem Biophys Res Commun 1987 149 672 679 10.1016/0006-291X(87)90420-7 2447882

[B7] Brange J Hansen JF Langkjaer L Markussen J Ribel U Sorensen AR Insulin analogues with improved absorption characteristics Horm Metab Res 1992 Supplement Series No 26 125 130 1490679

[B8] Hansen B The impact of the chemical synthesis of insulins on the development of new pharmaceuticals Akt Endokrin Stoffw 1990 11 212 214

[B9] Bähr M Kolter T Seipke G Eckel J Growth promoting and metabolic activity of the human insulin analogue [GlyA21,ArgB31,ArgB32] insulin (HOE 901) in muscle cells Eur J Pharmacol 1997 320 259 265 10.1016/S0014-2999(96)00903-X 9059862

[B10] Bennet RG Fawcett J Kruer MC Duckworth WC Hamel FG Insulin inhibition of the proteasome is dependent on degradation of insulin by insulin-degrading enzyme J Endocrinol 2003 177 399 405 10.1677/joe.0.1770399 12773120

[B11] Berti L Kellerer M Bossenmaier B Seffer E Seipke G Häring HU The long acting human insulin analog HOE 901: characteristics of insulin signalling in comparison to Asp(B10) and regular insulin Horm Metab Res 1998 30 123 129 10.1055/s-2007-978849 9566852

[B12] Boehm A Staiger H Hennige AM Haas C Machicao F Häring HU Effect of insulin detemir, compared to human insulin, on 3T3-LI adipogenesis Regul Pept 2008 151 160 163 10.1016/j.regpep.2008.05.005 18571747

[B13] Chisalita SI Arnqvist HJ Insulin-like growth factor 1 receptors are more abundant than insulin receptors in human micro- and macrovascular endothelial cells Am J Physiol Endocrinol Metab 2004 286 E 896 E 901 10.1152/ajpendo.00327.2003 14722023

[B14] Ciaraldi TP Carter L Seipke G Mudaliar S Henry RR Effects of the long-acting insulin analog insulin glargine on cultured human skeletal muscle cells: comparisons to insulin and IGF-1 J Clin Endocrinol Metab 2001 86 5838 5847 10.1210/jc.86.12.5838 11739448

[B15] Ciaraldi TP Phillips SA Carter L Aroda V Mudaliar S Henry RR Effects of the rapid-acting insulin analog glulisine on cultured human skeletal muscle cells: comparisons with insulin and insulin-like growth factor 1 J Clin Endocrinol Metab 2005 90 5551 5558 10.1210/jc.2005-1007 16030168

[B16] Eckardt K May C Koenen M Eckel J IGF-1 receptor signalling determines the mitogenic potency of insulin analogues in human smooth muscle cells and fibroblasts Diabetologia 2007 50 2534 2543 10.1007/s00125-007-0815-9 17898992

[B17] Erbel S Reers C Eckstein VW Proliferation of Colo-357 pancreatic carcinoma cells and survival of patients with pancreatic carcinoma are not altered by insulin glargine Diabetes Care 2008 31 1105 1111 10.2337/dc07-2015 18316392

[B18] Fawcett J Hamel FG Bennet RG Vajo Z Duckworth WC Insulin and analogue effects on protein degradation in different cell types-dissociation between binding and activity J Biol Chem 2001 276 11552 11558 10.1074/jbc.M007988200 11116143

[B19] Fawcett J Tsui BT Kruer MC Duckworth WC Reduced action of insulin glargine on protein and lipid metabolism: possible relationship to cellular hormone metabolism Metabolism 2004 53 1037 1044 10.1016/j.metabol.2004.02.013 15281015

[B20] Hamel FG Siford GL Fawcett J Chance RE Frank BH Duckworth WC Differences in cellular processing of AspB10 human insulin compared with human insulin and LysB28ProB29 human insulin Metabolism 1999 48 611 617 10.1016/S0026-0495(99)90059-8 10337862

[B21] Hansen BF Danielsen GM Drejer K Sörensen AR Wiberg FC Klein HH Lundemose AG Sustained signalling from the insulin receptor after stimulation with insulin analogues exhibiting increased mitogenic potency Biochem J 1996 315 271 279 8670118 10.1042/bj3150271 PMC1217182

[B22] Hansen BF Stidsen CE Glendorf T Lundby A Hegelund AC Bonnesen C Safety analysis of basal insulins: mitogenic potency and receptor binding. Abstract Diabetologia 2010 53 Suppl 1 S 22

[B23] Hennige AM Lehmann R Weigert C Moeschel K Schäuble M Metzinger E Lammers R Häring HU Insulin glulisine. Insulin receptor signalling characteristics in vivo Diabetes 2005 54 361 366 15677493 10.2337/diabetes.54.2.361

[B24] Hennige A Strack V Metzinger E Seipke G Häring HU Kellerer M Effects of new insulin analogues HMR1964(insulin glulisine) and HMR1423 on insulin receptors Diabetologia 2005 48 1891 1897 10.1007/s00125-005-1870-8 16052329

[B25] Kohn WD Micanovic R Myers SL pI-shifted insulin analogs with extended in vivo time action and favourable receptor selectivity Peptides 2007 28 935 948 10.1016/j.peptides.2007.01.012 17328992

[B26] Kurtzhals P Schaffer L Sorensen A Correlations of receptor binding and metabolic and mitogenic potencies of insulin analogs designed for clinical use Diabetes 2000 49 999 1005 10.2337/diabetes.49.6.999 10866053

[B27] Liefvendahl E Arnquist HJ Mitogenic effects of the insulin analogue glargine in malignant cells in comparison with insulin and IGF-1 Horm Metab Res 2008 40 369 374 10.1055/s-2008-1062739 18393172

[B28] Liu L Koenen M Seipke G Eckel J IGF-1 receptor-mediated signalling of the human insulin analogue HOE 901. Abstract Diabetologia 1997 40 Suppl 1 A 355

[B29] Liu S Li Y Liang Y Fan X Liang Q Yan L Insulin glargine and human insulin promote proliferation of human breast cancer cell line MDA-MB-231 independent of ERK pathway.Abstract Diabetes 2010 59 Supplement 1 Abstract.No.2488-PO

[B30] Liu S Li Y Liang Y Lin T High dose detemir inhibits proliferation of a human bladder cancer T24 cell line independent of Akt and ERK pathway. Abstract Diabetologia 2010 53 Suppl 1 S 305

[B31] Liu S Li Y Lin T Fan X Liang Y Heemann U High dose human insulin and insulin glargine promote T24 bladder cancer cell proliferation via PI3K-independent activation of Akt Diabetes Res Clin Pract 2011 91 177 182 10.1016/j.diabres.2010.11.009 21129803

[B32] Mayer D Shukla A Enzmann H Proliferative effects of insulin analogues on mammary epithelial cells Arch Physiol Biochem 2008 114 38 44 10.1080/13813450801900645 18465357

[B33] Mayer D Chantelau E Treatment with insulin glargine (Lantus^®^) increases the proliferative potency of the serum of patients with type-1 diabetes: a pilot study on MCF-7 breast cancer cells Arch Physiol Biochem 2010 116 73 78 10.3109/13813451003631439 20199195

[B34] Müller K Weidinger C Führer D Insulin glargine and insulin have identical effects on proliferation and phosphatidylinositol 3-kinase/AKT signalling in rat thyrocytes and human follicular cancer cells. Letter Diabetologia 2010 53 1001 1003 10.1007/s00125-010-1674-3 20179899

[B35] Rakatzi I Ramrath S Ledwig D Dransfeld O Bartels T Seipke G Eckel J A novel insulin analog with unique properties Diabetes 2003 52 2227 2238 10.2337/diabetes.52.9.2227 12941761

[B36] Rakatzi I Seipke G Eckel J [LysB3,GluB29] insulin: a novel insulin analog with enhanced ß-cell protective action Biochem Biophys Res Commun 2003 310 852 859 10.1016/j.bbrc.2003.09.090 14550282

[B37] Russo I Massucco P Mattiello L Cavalot F Anfossi G Trovati M Comparison between the effects of the rapid recombinant insulin analog aspart and those of human regular insulin on platelet cyclic nucleotides and aggregation Thromb Res 2002 107 31 37 10.1016/S0049-3848(02)00182-2 12413586

[B38] Russo I Massucco P Mattiello L Anfossi G Trovati M Comparison between the effects of the rapid recombinant insulin analog lispro (Lys B28, Pro B 29) and those of human regular insulin on platelet cyclic nucleotides and aggregation Thromb Res 2003 109 323 327 10.1016/S0049-3848(03)00255-X 12818257

[B39] Sandow J Seipke G In vitro pharmacology studies with insulin glargine and human insulin: IGF-1 receptor binding and thymidine incorporation. Abstract Diabetes 2001 50 Suppl 2 A 429

[B40] Sciacca L Cassarino MF Genua M Pandini G Le Moli R Squatrito S Vigneri R Insulin analogues differently activate insulin receptor isoforms and post-receptor signalling Diabetologia 2010 53 1743 53 10.1007/s00125-010-1760-6 20424816

[B41] Shukla A Insulin analogues: analysis of proliferative potency and characterisation of receptors and signalling pathways activated in human mammary epithelial cells. Dissertation Faculty of Natural Sciences 2008 Ruperto-Carola University of Heidelberg, Germany

[B42] Shukla A Grisouard J Ehemann V Hermani A Enzmann H Mayer D Analysis of signalling pathways related to cell proliferation stimulated by insulin analogs in human mammary epithelial cell lines Endocr Relat Cancer 2009 16 429 441 10.1677/ERC-08-0240 19153208

[B43] Shukla A Enzmann H Mayer D Proliferative effect of Apidra^® ^(insulin glulisine), a rapid acting insulin analogue on mammary epithelial cells Arch Physiol Biochem 2009 115 119 126 10.1080/13813450903008628 19480564

[B44] Slieker LJ Sundell KL In vitro analysis of Lys(B28),Pro(B29) human insulin (LY 275585): comparison to human insulin in terms of insulin and IGF-1 receptor binding, glucose uptake into adipocytes and thymidine incorporation into smooth muscle cells Unpublished report on file at Lilly Research Laboratories, Preclinical Pharmacology Report no.7 1994

[B45] Slieker LJ Brooke GS Di Marchi RD Modifications in the B10 and B26-30 regions of the B chain of human insulin alter affinity for the human IGF-1 receptor more than for the insulin receptor Diabetologia 1997 40 Suppl 2 S 54 S 61 10.1007/s001250051402 9248702

[B46] Sommerfeld MR Müller G Tschank G Seipke G Habermann P Kurrle R Tennagels N In vitro metabolic and mitogenic signaling of insulin glargine and its metabolites PLoS One 2010 5 3 e9540 10.1371/journal.pone.0009540 20209060 PMC2832019

[B47] Somwar R Sweeney G Ramlai T Klip A Stimulation of glucose and amino acid transport and activation of the insulin signalling pathways by insulin lispro in L6 skeletal muscle cells Clin Ther 1998 20 125 140 10.1016/S0149-2918(98)80040-4 9522110

[B48] Sorensen AR Stidsen CE Ribel U Nishimura E Sturis J Jonassen I Bouman SD Kurtzhals P Brand CL Insulin detemir is a fully efficacious, low affinity agonist at the insulin receptor Diabetes Obes Metab 2010 12 665 673 10.1111/j.1463-1326.2010.01206.x 20590743

[B49] Staiger K Staiger H Schweitzer MA Metzinger E Balletshofer B Häring HU Kellerer M Insulin and its analogue glargine do not affect viability and proliferation of human coronary artery endothelial and smooth muscle cells Diabetologia 2005 48 1898 1905 10.1007/s00125-005-1874-4 16078017

[B50] Staiger K Hennige AM Staiger H Häring HU Kellerer M Comparison of the mitogenic potency of regular human insulin and its analogue glargine in normal and transformed human breast epithelial cells Horm Metab Res 2007 39 65 67 10.1055/s-2007-957352 17226117

[B51] Stammberger I Seipke G Bartels Insulin glulisine - a comprehensive preclinical evaluation Int J Toxicol 2006 25 25 33 10.1080/10915810500488379 16510354

[B52] Tews D Werner U Eckel J Enhanced protection against cytokine- and fatty acid-induced apoptosis in pancreatic beta cells by combined treatment with glucagons-like peptide-1 receptor agonists and insulin analogues Horm Metab Res 2008 40 172 180 10.1055/s-2008-1042426 18348079

[B53] Volund A Brange J Drejer K Jensen I Markussen J Ribel U Sorensen AR Schlichtkrull J In vitro and in vivo potency of insulin analogues designed for clinical use Diabetic Med 1991 8 839 847 10.1111/j.1464-5491.1991.tb02122.x 1663018

[B54] Wada T Azegami M Sugiyama M Tsuneki H Sasaoka T Characteristics of signalling properties mediated by long-acting insulin analogue glargine and detemir in target cells of insulin Diabetes Res Clin Pract 2008 81 269 277 10.1016/j.diabres.2008.05.007 18585815

[B55] Warnken M Reitzenstein U Sommer A Fuhrmann M Mayer P Enzmann H Juergens UR Racke K Characterization of proliferative effects of insulin, insulin analogues and insulin-like growth factors-1 (IGF-1) in human lung fibroblasts Naunyn-Schmied Arch Pharmacol 2010 382 511 524 10.1007/s00210-010-0561-2 20924562

[B56] Weinstein D Simon M Yehezkel E Laron Z Werner H Insulin analogues display IGF-1-like mitogenic and anti-apoptotic activities in cultured cancer cells Diabetes Metab Res Rev 2009 25 41 49 10.1002/dmrr.912 19145584

[B57] Yehezkel E Weinstein D Simon M Sarfstein R Laron Z Werner H Long-acting insulin analogues elicit atypical signalling events mediated by the insulin receptor and insulin-like growth factor-1 receptor Diabetologia 2010 53 2667 2675 10.1007/s00125-010-1899-1 20835859

[B58] EMEA Scientific Discussion of insulin aspart (NovoRapid^®^) 2010 http://www.ema.europa.eu/docs/en_GB/document_library/EPAR_-_Scientific_Discussion/human/000258/WC500030369.pdf

[B59] EMEA Scientific Discussion of insulin detemir (Levemir^®^) 2010 http://www.ema.europa.eu/docs/en_GB/document_library/EPAR_-_Scientific_Discussion/human/000528/WC500036658.pdf

[B60] EMEA Scientific Discussion of insulin glargine (Lantus^®^) 2010 http://www.ema.europa.eu/docs/en_GB/document_library/EPAR_-_Scientific_Discussion/human/000284/WC500036075.pdf

[B61] EMEA Scientific Discussion of insulin glulisine (Apidra^®^) 2010 http://www.ema.europa.eu/docs/en_GB/document_library/EPAR_-_Scientific_Discussion/human/000557/WC500025246.pdf

[B62] EMEA Scientific Discussion of insulin lispro (Humalog^®^) http://www.ema.europa.eu/docs/en_GB/document_library/EPAR_-_Scientific_Discussion/human/000088/WC500050329.pdf accessed 17.11.2010

[B63] Conover AC Clarkson JT Bale LK Physiological concentrations of insulin induce cellular desensitization to the mitogenic effect of insulin-like growth factor 1 Diabetes 1994 43 1130 1137 10.2337/diabetes.43.9.1130 8070613

[B64] Khokher MA Dandona P Comparison of biologic potency of human insulin (recombinant DNA) and purified porcine insulin (PPI) on the rat and human adipocyte lipogenesis model Diabetes Care 1982 5 Suppl 2 102 103 6765517 10.2337/diacare.5.2.s102

[B65] EMEA Committee for proprietary medicinal products CPMP:Points to consider document on the non-clinical assessment of the carcinogenic potential of insulin analogues Internet search: CPMP/SWP/372/01 2001 Issued in London 15

[B66] Hansen BF Insulin analogues with increased mitogenic potency- are they safe? Horm Metab Res 2008 40 431 433 10.1055/s-2008-1062740 18418813

[B67] Belfiore A Frasca F Pandini G Sciacca L Vigneri R Insulin receptor isoforms and insulin receptor/insulin-like growth factor receptor hybrids in physiology and disease Endocrine Reviews 2009 30 586 623 10.1210/er.2008-0047 19752219

[B68] Zhang C Hao L Xiao Y Ge H Zhu Z Luo Y Zhang Y Zhang Y Elevated IGFIR expression regulating VEGF and VEGF-C predicts lymph node metastasis in human colorectal cancer BMC Cancer 2010 10 184 10.1186/1471-2407-10-184 20459642 PMC2873398

[B69] Vigneri R Squatrito S Sciacca L Insulin and its analogs: actions via insulin and IGF receptors Acta Diabetol 2010 47 271 278 10.1007/s00592-010-0215-3 20730455

[B70] Masur K Vetter C Hinz A Tomas N Henrich H Niggemann B Zänker KS Diabetogenic glucose and insulin concentrations modulate transcriptom and protein levels involved in tumour cell migration, adhesion and proliferation Br J Cancer 2011 104 345 352 10.1038/sj.bjc.6606050 21179032 PMC3031898

[B71] Leroy EC Moore JH Hu C Martinez ME Lance P Duggan D Thompson PA Genes in the insulin and insulin-like growth factor pathway and odds of metachronous colorectal neoplasia Hum Genet 2011 129 503 512 10.1007/s00439-010-0942-0 21221997

[B72] Varewijck AJ Goudzwaard JA Brugts MP Lamberts SWJ Hofland LJ Janssen JAMJL Insulin glargine is more potent in activating the human IGF-1 receptor than human insulin and insulin detemir Growth Horm IGF Res 2010 20 427 431 10.1016/j.ghir.2010.10.002 21055982

[B73] Smeeton F Shojaee Moradie F Jones RH Westergaard L Haahr H Umpleby AM Russell-Jones DL Differential effects of insulin detemir and neutral protamine Hagedorn (NPH) insulin on hepatic glucose production and peripheral glucose uptake during hypoglycaemia in type 1 diabetes Diabetologia 2009 52 2317 2323 10.1007/s00125-009-1487-4 19707744

[B74] Hennige AM Sartorius T Tschritter O Preissl H Fritsche A Ruth P Häring HU Tissue selectivity of insulin detemir action in vivo Diabetologia 2006 49 1274 1282 10.1007/s00125-006-0192-9 16570163

